# Investigation into the Impact of Hollow Cylinder Inner Diameter on the Mechanical Properties of Biomimetic Voronoi Scaffolds

**DOI:** 10.3390/jfb17080369

**Published:** 2026-07-30

**Authors:** Zainab Alknery, Ali Arab

**Affiliations:** 1 Department of Aircraft Engineering, College of Engineering, Salahaddin University-Erbil, Erbil 44001, Kurdistan Region, Iraq; 2 R&D Department, Bermar International Ltd., Ipswich IP5 3RG, UK

**Keywords:** mechanical properties, energy absorption, porous biomaterials, biomimetic scaffold, hollow voronoi structure, stereolithography (SLA)

## Abstract

The current study investigates the mechanical performance of the hollow cylindrical scaffolds with varying inner radii designed for bone tissue engineering. The biomimetic Voronoi-based structures with distinct inner diameters were manufactured using the stereolithography (SLA) technique and eco-friendly plant-based resin. The experimental results revealed that increasing the inner radius significantly reduces the mechanical properties of the biological scaffolds. Conversely, designs with a smaller inner diameter exhibited higher resistance to compressive stress and superior energy absorption. This work provides vital insights into constructing functional biodegradable supports which balance structural integrity with biological compatibility by optimising specific geometric parameters. These discoveries are intended to improve the efficiency of synthetic scaffolds utilised for medical regeneration and repair of fractures.

## 1. Introduction

Bone tissue regeneration in orthopaedics presents substantial hurdles, particularly for large injuries that exceed crucial healing thresholds. Conventional treatments, like autografts and allografts, have limitations, including donor site morbidity and immunological rejection. As a result, bone tissue engineering (BTE) has evolved as a novel approach that uses cells, scaffolds, and biochemical agents to promote tissue regeneration [1]. In BTE, 3D porous scaffolds serve as temporary extracellular matrices that offer mechanical support and promote cell adhesion and proliferation. An effective scaffold should have high porosity, interlinked pores, and appropriate mechanical properties to resemble natural bone tissue. Porous structures are vital for nutrient transfer, vascularisation, and osseointegration, which are crucial for successful rapid regeneration [2,3]. Furthermore, the biological potential for tissue ingrowth and mechanical stability must be carefully balanced for these implants to be successful [4]. Although numerous currently available bone scaffolds are manufactured from materials with high mechanical strength, ensuring adequate biological performance while maintaining structural integrity remains a significant challenge [5].

Prior to recent advances in computational design and additive manufacturing, scaffold structures were largely limited to simple, regular geometries because conventional software could not efficiently generate complex porous structures. Early studies mainly investigated the mechanical behaviour of regular lattice structures under compression. For example, Ahmadi et al. evaluated the compressive performance of six regular scaffold geometries [6], while Kantaros et al. compared simple cubic, face-centred cubic, and ring-centred structures to examine the influence of geometry on mechanical properties [7]. Recent developments in parametric modelling and additive manufacturing have enabled the fabrication of more complex biomimetic architectures. Among these, Voronoi-based scaffolds have attracted considerable attention because they closely resemble the irregular and stochastic morphology of natural trabecular bone. Unlike conventional periodic lattices, Voronoi structures provide a heterogeneous pore distribution that can improve stress distribution, reduce stress shielding, and promote tissue ingrowth while maintaining suitable mechanical performance [8,9,10,11,12]. More recently, hollow scaffold designs have emerged as an effective strategy for further improving scaffold performance. By introducing a hollow core within the struts, the wall thickness and structural volume fraction can be adjusted without substantially changing the overall scaffold porosity. This provides greater flexibility in balancing lightweight design, mechanical strength, and biological functionality. In particular, geometric parameters such as the inner-to-outer radius ratio directly influence the load-bearing wall thickness and, consequently, the mechanical response of the scaffold [13,14]. Despite these advances, most previous studies have focused on pore geometry, porosity distribution, or lattice topology. The influence of the inner radius of hollow cylindrical struts within Voronoi scaffolds has received little attention, especially through experimental investigation. Since the inner radius directly controls the wall thickness, it is expected to influence load transfer, stress concentration, stiffness, and energy absorption. Although a reduction in mechanical strength with increasing inner radius is anticipated, the objective of this study extends beyond confirming this trend. Instead, it is to identify the balance between lightweight design and compressive load-bearing capacity by establishing practical design limits for hollow Voronoi scaffolds with different inner radii.

On the other hand, the selection of material is crucial for scaffold effectiveness [15]. In recent years, biocompatible and biodegradable polymers, especially bio-based photopolymers, have achieved popularity because of their environmental friendliness, being widely available in nature [16], and appropriateness for SLA manufacturing. In addition, these materials could be treated into extremely complex porous architectures while still retaining appropriate mechanical characteristics for biomedical purposes and aerospace [17,18]. Recent developments in additive manufacturing (AM) techniques have made it possible to fabricate sophisticated porous scaffolds with precise geometry, pore size, and internal structure [19]. Compared with conventional manufacturing techniques, AM enables the production of patient-specific structures with tailored mechanical and biological characteristics, making it a powerful tool in bone tissue engineering. Among the available AM technologies, stereolithography (SLA) has attracted considerable interest because of its high fabrication accuracy, excellent surface quality, and ability to produce intricate porous geometries with interconnected pore networks [20,21], such as interconnected porosity in lattice structures, with minimal support systems—a major benefit for tissue engineering and bone scaffold purposes [22]. For example, Chen et al. developed 3D-printed bone scaffolds using the stereolithography technique by mimicking the complex, high-porosity structure of a real loofah. Their findings indicate that using SLA 3D printing to mimic complex structures successfully restores the metabolic function of bone in fractures [23].

While conventional research has comprehensively documented the bulk features of regular geometry of scaffolds, there is an increasing interest in hollow cylindrical structures for their ability to optimise grafting environments and prevent inflammation [6]. Nonetheless, most existing studies on open-cell Voronoi structures are restricted to solid strut typologies, and a critical knowledge gap remains regarding the specific influence of the inner radius in hollow cylindrical configurations on mechanical properties. Thus, investigating this geometric transition is crucial in order to separate structural weight and biological mass transport parameters from global porosity boundaries. The present work investigated the effect of the inner radius of hollow cylindrical Voronoi-based scaffolds and identified the critical balance between increasing the hollow inner radius and maintaining adequate compressive stability. Scaffolds with three variations in inner radius were manufactured via SLA 3D printing and assessed through experimental compressive testing. Consequently, the obtained parametric investigation findings provide valuable insights into the optimal design of biomimetic scaffolds for bone tissue engineering applications, especially non-cortical and trabecular bone reconstruction, allowing the scaffold to operate within the lower stiffness domains of native cancellous bone while avoiding stress shielding.

## 2. Materials and Methods

### 2.1. Materials

The commercially promoted plant-based resin (ANYCUBIC 3D technology company, Shenzhen, Guangdong, China) with a bending strength of 40–60 MPa and tensile strength of 35–55 MPa was used to manufacture the three samples [24]. Plant-based resin is a UV-curable photopolymer used in stereolithography (SLA) 3D printing because it is eco-friendly, free of VOCs and toxic chemicals, optimised for minimising shrinkage and enhancing printing quality, biocompatible, and sensitive to 365–405 nm UV light [25]. This high-fidelity SLA matrix allowed precise mechanical analysis without the thermal distortions that are seen in other techniques. Consequently, providing a structural blueprint easily transferable to biocompatible polymers.

### 2.2. Scaffolds Design

**Figure 1:**
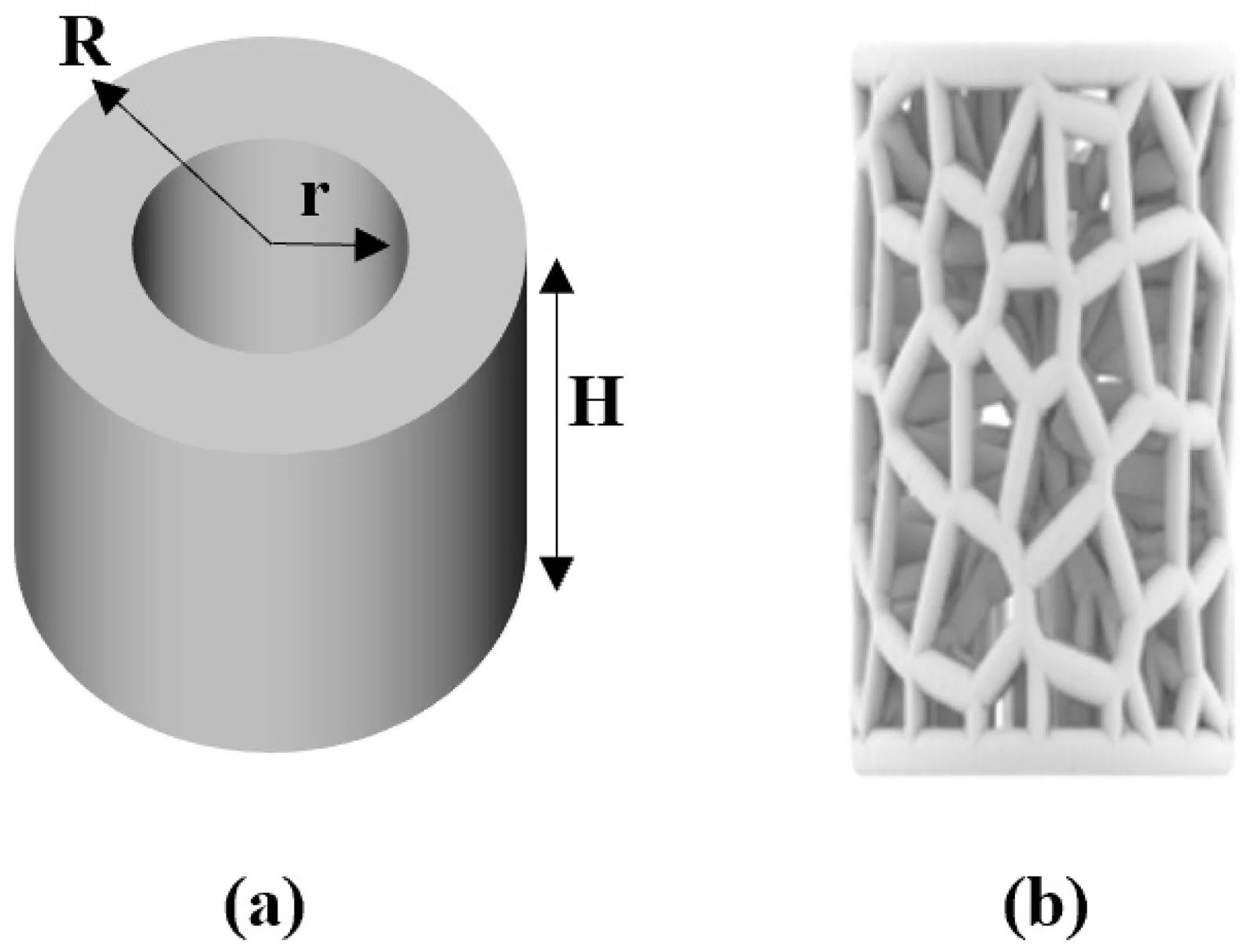
An illustration of: (**a**) hollow Voronoi structures (HVSs) and (**b**) cylinder details.

**Figure 2:**
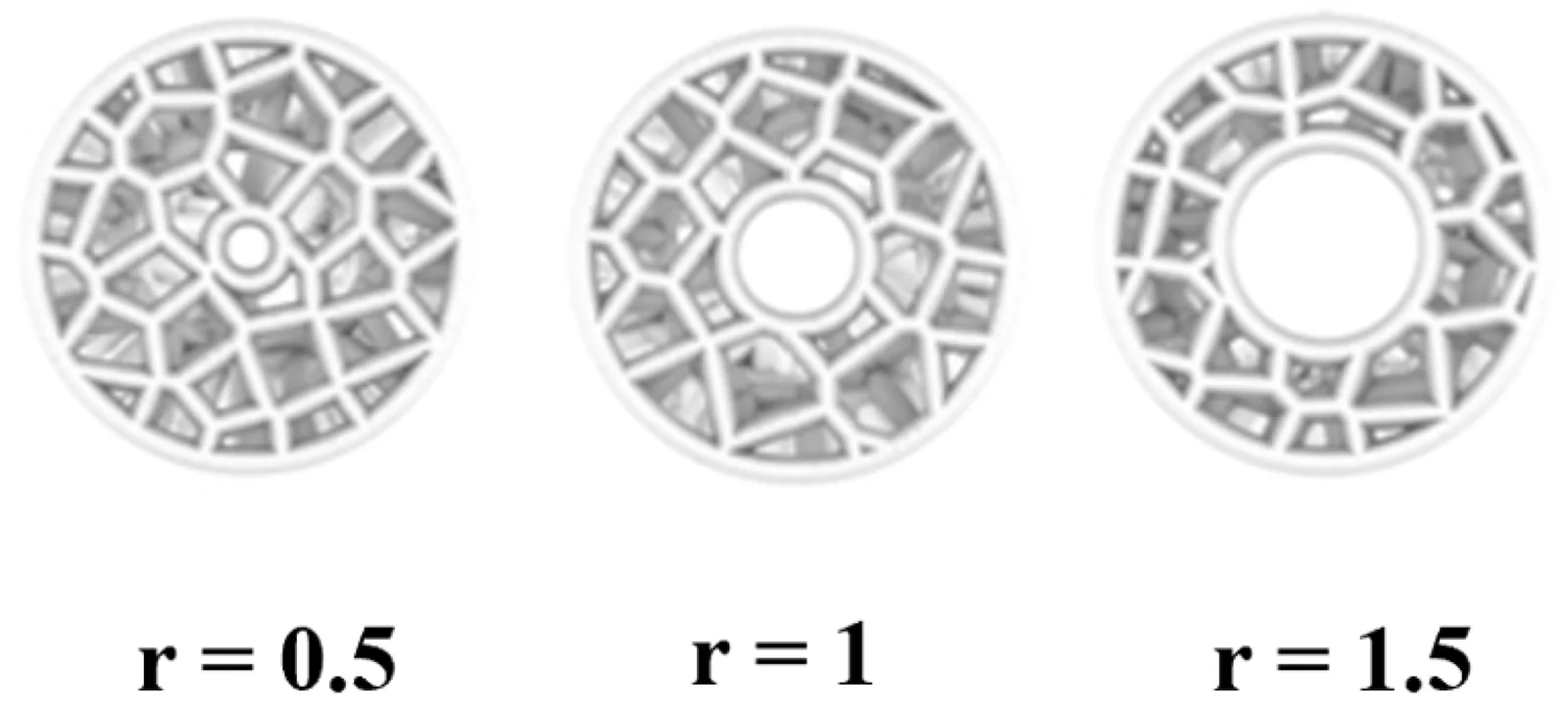
The top view of the three designed models.

**Figure A1:**
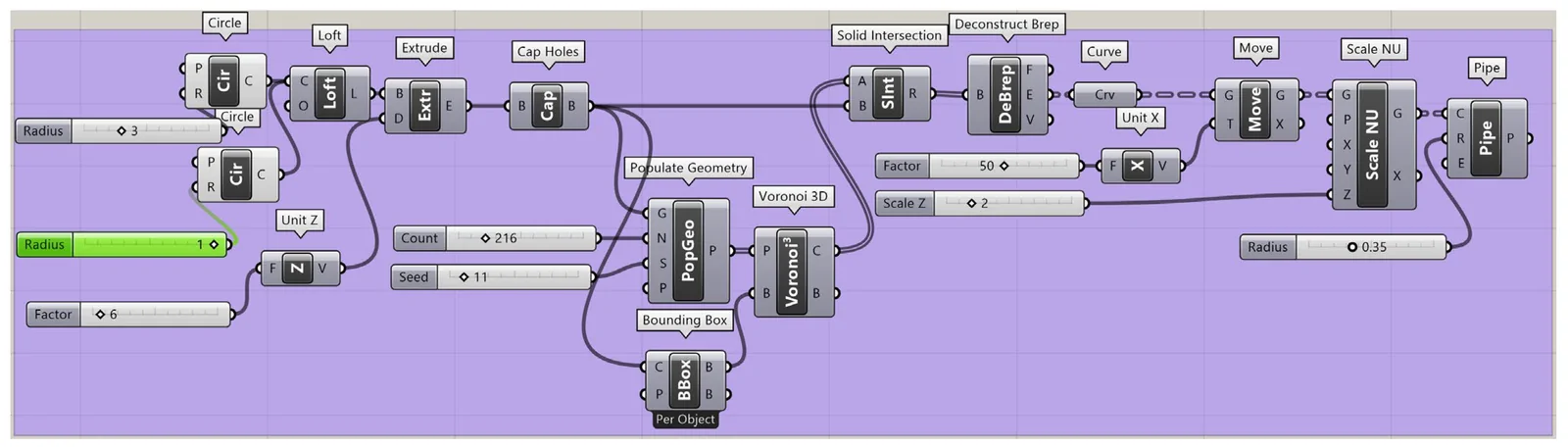
The Grasshopper file for the hollow cylinder—Voronoi structure.

In this work, the Rhinoceros 7 (Grasshopper) software (Robert McNeel & Associates (Seattle, WA, Usa; URL: https://www.rhino3d.com) was used to design three models of scaffolds with different inner radii of the hollow cylinder to obtain various levels of area (r = 0.5, 1.0, and 1.5 mm) for Cylinder I, Cylinder II, and Cylinder III, respectively, in order to evaluate the spatial core-to-surface transition effects. The height was fixed at H = 12 mm with an outer diameter of D = 6.54 mm according to the ASTM D695 standards to achieve an aspect ratio of 1.83 to prevent the premature macroscopic buckling. All types of scaffolds were modelled as a 3D cylinder with a 95 mm^3^ volume, a thickness of 0.1 mm and different numbers of seeds as shown in Table 1. The parametric Grasshopper script dynamically balances the density of seed and wall configurations to maintain a uniform relative density of about 0.37 (37%) with a constant total volume across all the models. Consequently, Cylinder I uses fewer seed points due to its minimal core void, whereas Cylinders II and III require higher seed counts to compensate for the material removed by the expanded inner radii, keeping the physical weight nearly unchanged. The microstructures of bones are diverse, while the inner part is a cancellous bone with porosity of about 50–90%, which is chiefly composed of trabecular bone [26]; therefore, we set the porosity around 63%. Such scaffold designs are based on hollow Voronoi structures (HVSs), the details of which are indicated in Figure 1a,b. HVSs were designed as a cylinder with an axial hole in the middle with different inner radii, as indicated in Figure 2. The process began by distributing several points randomly in the 3D cylindrical elongated Voronoi structure to obtain a specific volume with defined dimensions. The cylinder wall consists of a 3D Voronoi lattice structure formed by interconnected solid struts, creating irregular polygonal pores that allow fluid flow, while the central core forms a hollow cavity. This cavity forms a continuous, open-ended axial channel that increases the internal void space to facilitate fluid transport and nutrient exchange. Figure A1 shows the parametric design workflow and the plugin configuration deployed for scaffold generation. The porosity was determined by the gravimetric method as shown in Equations (1) and (2) [27,28,29,30].

(1) $$\rho_{\text{scaffold}}=\text{mass}/\text{volume}$$

(2) $$\text{Total}\;\text{porosity}=1-(\rho_{\text{scaffold}}/\rho_{\text{material}})$$

where $\rho_{\text{material}}$ is the density of the material and $\rho_{\text{scaffold}}$ is the apparent density of the scaffold.

### 2.3. Three-Dimensional Printing

**Figure 3:**
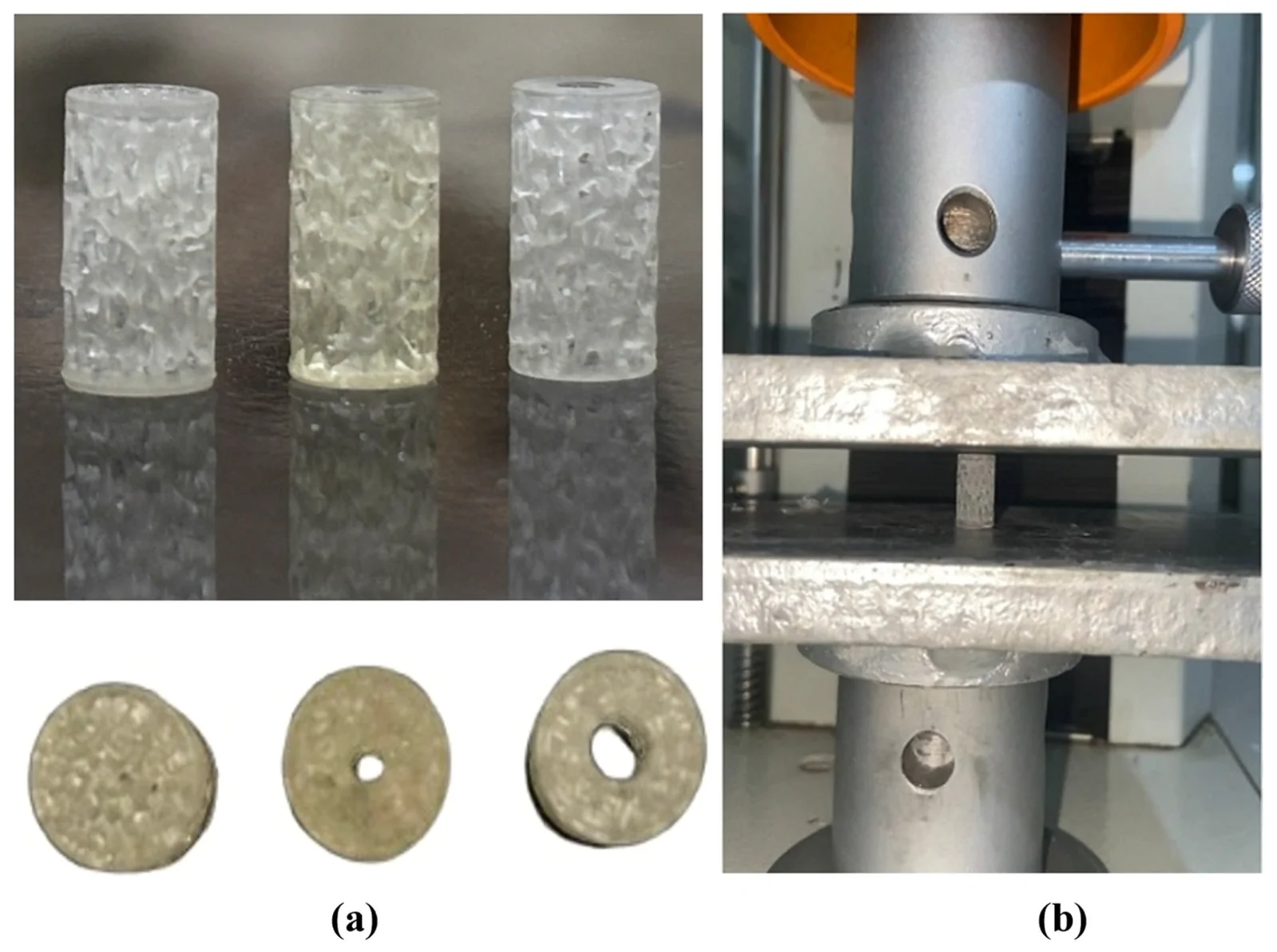
(**a**) 3D printed scaffolds showing the front and top view and (**b**) GOTECH AI-3000 universal testing machine.

Three models of samples were saved as an STL file before being sent for printing. The SLA technique was used to print the samples due to its precision in printing geometric shapes with complex details [31] as demonstrated in Figure 3a. The SLA printer (ELFIN 3 Mini, NovaMaker, Creality, Shenzhen, China) includes a build platform, which is where the part is made. A resin tank is located beneath, and clear glass allows the UV laser to cure the resin. The parameters were identified by NovaMaker (v2.8, NOVA 3D) printing control software at a layer thickness of 0.05 mm, (118.8 × 66.8 × 150) mm^3^ printing size, a normal layer exposure time of 2.5 s, a bottom layer exposure of 30 s, a lift distance of 6 mm, a lifting speed of 50 mm/min, and a 90° printing angle with ±0.1 mm model tolerance.

### 2.4. Mechanical Test

The uniaxial compression tests were carried out on three Voronoi models with low-density samples using a GOTECH AI-3000 universal testing apparatus (Gotech Testing Machines Inc., Taichung, Taiwan) with a total height of 1570 mm, as shown in Figure 3b. The speed remained steady at 5 mm/min, and the test was conducted in a quasi-static environment with a strain rate of 0.001/sec according to the ASTM D695 standard for rigid plastics and applied to the manufactured cellular structures [32]. The applied load and displacement were measured by a digital board, and each sample was compressed at least four times (*n* = 4). The stress–strain curve was then plotted using an average of many curves. The apparent stress and apparent strain can be calculated from Equation (3) and Equation (4), respectively.

(3) $$\sigma^{*}=F/A=F/\pi R^{2}$$

(4) $$\varepsilon^{*}=∆L/L_{i}=\frac{\left(L_{i}-L_{f}\right)}{L_{i}}$$

where R is the radius of the scaffolds in mm, $L_{i}$ is the initial length of the porous scaffold in mm, $L_{f}$ is the length of the scaffold after the deformation in mm, and ∆L represents the compressive displacement (mm), based on the concept that the apparent stress is constant over the cross-sectional area and throughout the gauge length.

Additionally, the apparent stress and apparent strain are determined by gradually applying a load to a test specimen and measuring the subsequent deformation. Numerous properties of a material are revealed through this curve, including the apparent Young’s modulus $E^{*}$, stiffness $S^{*}$, and ultimate strength US. In the present study, the standard approach was utilised. The engineering stress can be estimated by dividing the applied load by the cross-sectional area of the sample (see Equation (3)). In order to compare the mechanical properties of the models, the apparent Young’s modulus and stiffness were calculated from Equation (5) and Equation (6), respectively, while ultimate strength could be determined precisely from the apparent stress–strain curve.

(5) $$E^{*}=\sigma^{*}/\varepsilon^{*}$$

(6) $$S^{*}=F/∆L$$

### 2.5. Energy Absorption Evaluation

Energy absorption is an important characteristic in biomedical engineering, particularly in scaffold construction in the event of a bone fracture. Because of differences in geometrical designs between samples, energy absorption was measured to compare their ability to absorb energy. The Total energy Absorption (TEA) is obtained from the area under the load–displacement curve using Equation (7) [33]:

(7) $$TEA=\int P_{ave}d_{s}\equiv P_{ave}(d_{f}-d_{i})$$

where $P_{ave}$ is the mean crushing force, $d_{i}$ is the initial crushing distance, and $d_{f}$ is the final crushing distance. Commonly, TEA is measured in kJ according to the SI unit. Since the proposed models were designed to be used to manufacture the biological scaffolds, they require a light weight. As a result, it is necessary to calculate the specific energy absorption (SEA) of the models. SEA is defined as the total energy absorbed divided by the mass of the sample, with the unit of kJ/kg [34]. Consequently, this can be determined based on Equation (8):

(8) SEA=TEA/MASS

### 2.6. Statistical Analysis

The experimental data are presented as ±standard deviation (*n* = 4). To assess the statistical significance of the geometric changes on the mechanical properties of the Voronoi scaffolds, a one-way analysis of variance (ANOVA) was performed using Microsoft Excel 2021 (Microsoft Crop., Redmond, WA, USA). In all the graphical plots, generated using Microsoft Excel 2021 and edited via Microsoft PowerPoinr 2021, statistical significance between the scaffold groups is denoted by a single asterisk notation (*) based on the calculated p-values.

## 3. Results and Discussion

### 3.1. Mechanical Properties

Table 2 shows the mechanical properties of the various models. It is clear that Cylinder I has the highest apparent Young’s modulus of 201 MPa, stiffness of 368.4 N/mm, and ultimate strength of about 22 MPa. Followed by Cylinder II with 142 MPa Young’s modulus, 286.9 N/mm of stiffness, and ultimate strength of 16.3 Mpa, while the lower mechanical properties of Young’s modulus, stiffness, and ultimate strength were exhibited by Cylinder III of about 42 MPa, 42.9 N/mm, and 4 MPa, respectively. Furthermore, the results show a strong association between the inner radius (r) of the hollow Cylinder components in Voronoi-based scaffolds and their mechanical properties. As the inner radius increased from 0.5 mm to 1.5 mm for Cylinder I and Cylinder III, respectively, all the measured mechanical parameters showed a sudden drop. The apparent Young’s modulus (E*) declined from 201 MPa for Cylinder I to 42 MPa for Cylinder III, indicating a roughly 80% reduction in structural stiffness. The same trend was seen in ultimate strength (US), which dropped from 22 MPa in Cylinder I to 4 MPa in Cylinder III.

**Figure 4:**
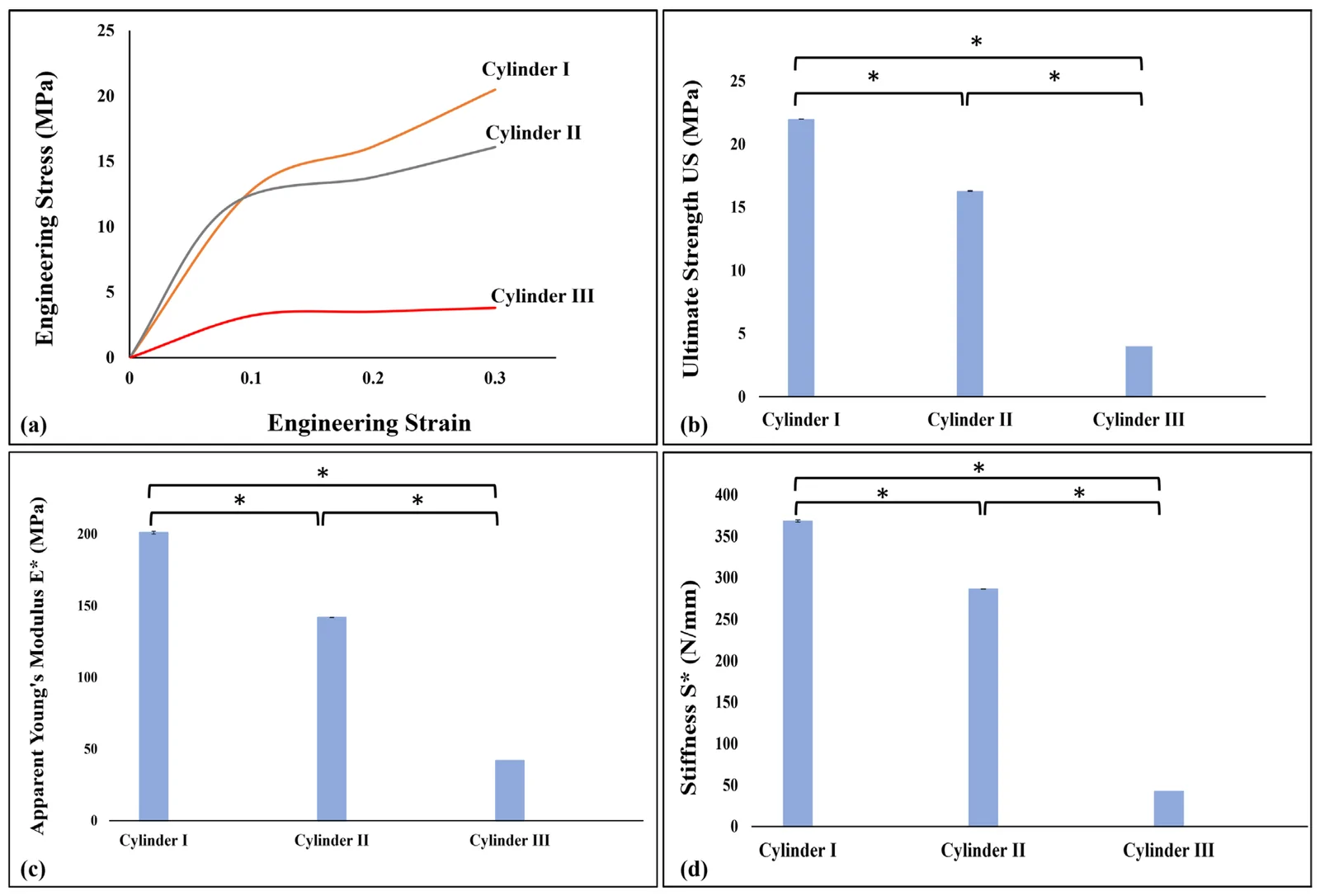
Experimental mechanical evaluation of three Voronoi structures under compressive loading: (**a**) apparent stress–strain curve; (**b**) ultimate strength (US); (**c**) apparent Young’s modulus (E*); and (**d**) stiffness (S*). Note: Error bars represent the standard deviation of *n* = 4 tested samples. Statistical Markers: a single asterisk notation (*) denotes a statistically significant difference (*p* < 0.05). Designs with differing letters are statistically distinct.

On the other hand, Figure 4a presents the apparent compressive stress–strain curves of the three cylindrical scaffold structures. The compression test was repeated four times for each design, and the average values are reported. Figure 4b–d summarise the average ultimate stress (US), apparent Young’s modulus (E*), and stiffness (S*), with error bars indicating the standard deviation. Inevitably, the small error bars represent good repeatability and consistency of the experimental results. Also, a one-way ANOVA statistical analysis was implemented to evaluate the differences in the mechanical properties among the three cylindrical scaffold designs. The mechanical properties across Cylinder I, Cylinder II, and Cylinder III showed an extremely statistically significant difference (F(2, 9)=167,206.2,  p<0.001). These notable differences are further supported by the standard deviation error bars demonstrated in the comparative bar charts. All three designs demonstrated the typical compressive behaviour of Voronoi (cellular) structures. The response began with a nearly linear elastic region, followed by a plateau region as the cellular network progressively deformed. It is worth noting that a similar deformation trend has been reported by Chao et al. [35], who explained that slight nonlinear behaviour at the beginning of compression could result from the initial contact between the indenter and the porous sample. Comparable stress–strain behaviour has also been reported for TPMS scaffolds by Zhang et al. [36]. Despite exhibiting similar deformation mechanisms, significant differences were observed in the apparent stress values among the investigated designs. Cylinder I (r = 0.5 mm) achieved an apparent stress of approximately 20.5 MPa at a strain of 0.3, whereas Cylinder II (r = 1.0 mm) and Cylinder III (r = 1.5 mm) reached only 16.1 MPa and 3.8 MPa, respectively, at the same strain level. The same trend is clearly confirmed by the bar charts in Figure 4b–d. Cylinder I achieved the highest ultimate stress, elastic modulus, and stiffness, whereas Cylinder III consistently produced the lowest values.

The reduction in mechanical performance with increasing inner radius is mainly due to the thinner hollow strut walls. As the inner radius increases, the wall thickness decreases, reducing the stiffness and load-carrying capacity of the cylindrical Voronoi scaffold. The thinner struts are more likely to bend, buckle, and develop local stress concentrations at the junctions where many struts meet. Similar observations were reported by Lu et al. [37], who found that larger internal voids reduce the mechanical efficiency of spatial Voronoi cellular structures. Likewise, Jang et al. [38] reported that thinner scaffold walls may improve biological performance but often reduce mechanical strength. Alemayehu and Todoh [39] found that increasing the pore size in Voronoi-inspired biomimetic structures reduced structural stability and overall load-bearing capacity. The results obtained in the present study further support previous findings reported by Seo et al. [40], who identified strut geometry as one of the most influential parameters governing the compressive performance of 3D-printed Voronoi trabecular structures. The marked reduction in stiffness observed in Cylinder III highlights the importance of improving the inner radius to achieve a balance between mechanical strength and scaffold functionality. Recent studies have also highlighted the importance of geometric optimisation in porous scaffolds [41]. Therefore, while a larger inner radius may enhance cell migration and nutrient transport, excessive wall thinning significantly weakens the scaffold under compressive loading.

Table 3 compares the mechanical properties of the proposed hollow Voronoi scaffolds with recently reported bone tissue engineering scaffolds, where key structural parameters, including porosity and relative density, are presented alongside the experimental compressive properties to provide a direct comparison. As shown, the proposed hollow Voronoi scaffolds exhibited mechanical behaviour that differed from the scaffold designs reported in the literature. Cylinder I and Cylinder II achieved ultimate compressive strengths of 22 MPa and 16.3 MPa, respectively, which are higher than most polymer and bioceramic scaffolds with similar or even greater porosity. For instance, at a porosity of about 65%, the proposed hollow scaffolds outperformed the Diamond (4–4.2 MPa) and Gyroid (5.5 MPa) structures reported by Diez-Escudero et al. [42], as well as the TPMS scaffolds developed by Guo et al. [43] and the I-WP structures reported in Xu et al. [44]. This improvement is mostly attributed to the hollow Voronoi structure, which distributes the applied load more effectively and delays structural collapse during compression. However, the apparent Young’s modulus (E*) demonstrated a different trend. The bioceramic scaffolds reported by Maevskaia et al. [45] exhibited much higher stiffness (700–3400 MPa) because they were manufactured from sintered hydroxyapatite (HAp), a material with inherently high rigidity.

In contrast, the plant-based resin used in the current study provided a lower apparent Young’s modulus ranging from 42 to 201 MPa. At a similar porosity level (63–65%), these values are comparable to those reported for polymer-based Diamond and Gyroid scaffolds (85–135 MPa) Diez-Escudero et al. [42]. This reveals that the proposed Hollow Voronoi scaffolds produce bone-like compliance while maintaining excellent mechanical performance. The results highlight the importance of local density distribution. Castro et al. [46] have reported that regions with lower porosity and higher local density enhance stiffness and increase the load-carrying capacity of cellular structures. In the proposed hollow Voronoi scaffolds, variations in cell connectivity and local density help redistribute the applied stress more effectively. The denser districts support the applied load, while the less dense regions deform through controlled strut bending during compression. As a result, the proposed design achieves a good balance between mechanical strength and structural compliance, making it a promising scaffold structure for bone tissue engineering applications.

**Figure 5:**
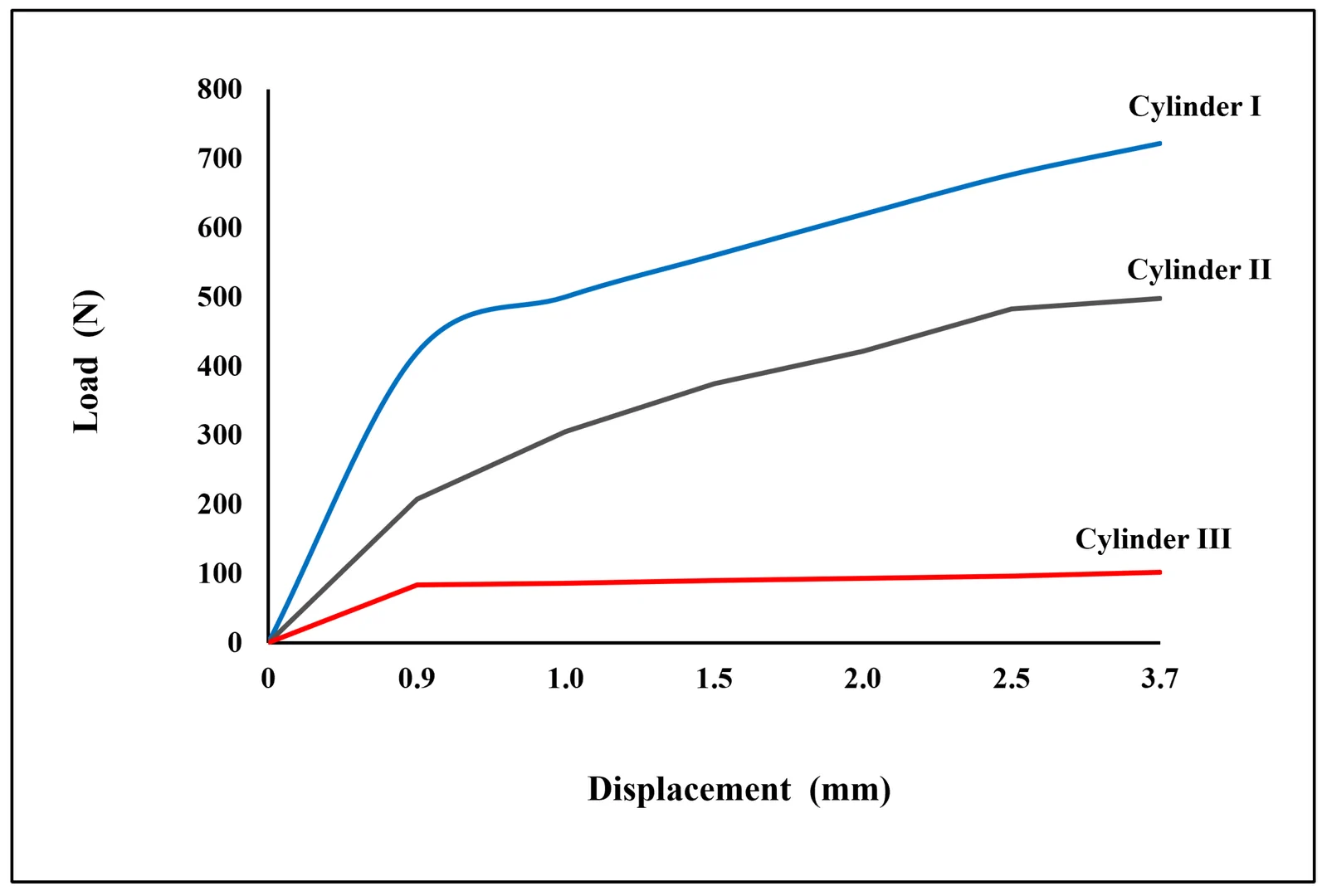
Load–displacement curves of three Voronoi structures.

In addition, the load–displacement curve is illustrated in Figure 5. The slope of the load–displacement curve can be used to directly evaluate each structure’s stiffness; steeper slopes indicate stronger resistance to deformation. Among the tested designs, Cylinder I displayed the highest structural stiffness, displaying higher resistance to compressive loading than the other configurations. Interestingly, despite reaching failure at a similar displacement of roughly 3.7 mm, Cylinder I and Cylinder III had very different stiffness values, measuring 368.4 N/mm and 42.9 N/mm, respectively. In comparison, Cylinder II failed at a lesser displacement of about 2 mm while maintaining reasonably high rigidity. These findings suggest that the scaffold’s deformation behaviour is influenced not only by its overall shape, but also by the wall thickness of the hollow cylindrical struts and their ability to withstand local instability processes. The observed mechanical behaviour can be explained using the Gibson–Ashby theory for cellular solids [47], which establishes a direct relationship between the mechanical properties of porous structures and their relative density. According to this theory, the elastic modulus scales with relative density according to Equation (9).

(9) $$E/E_{s}=C_{1}{\left(\rho /\rho_{s}\right)}^{n}$$

### 3.2. Energy Absorption

**Figure 6:**
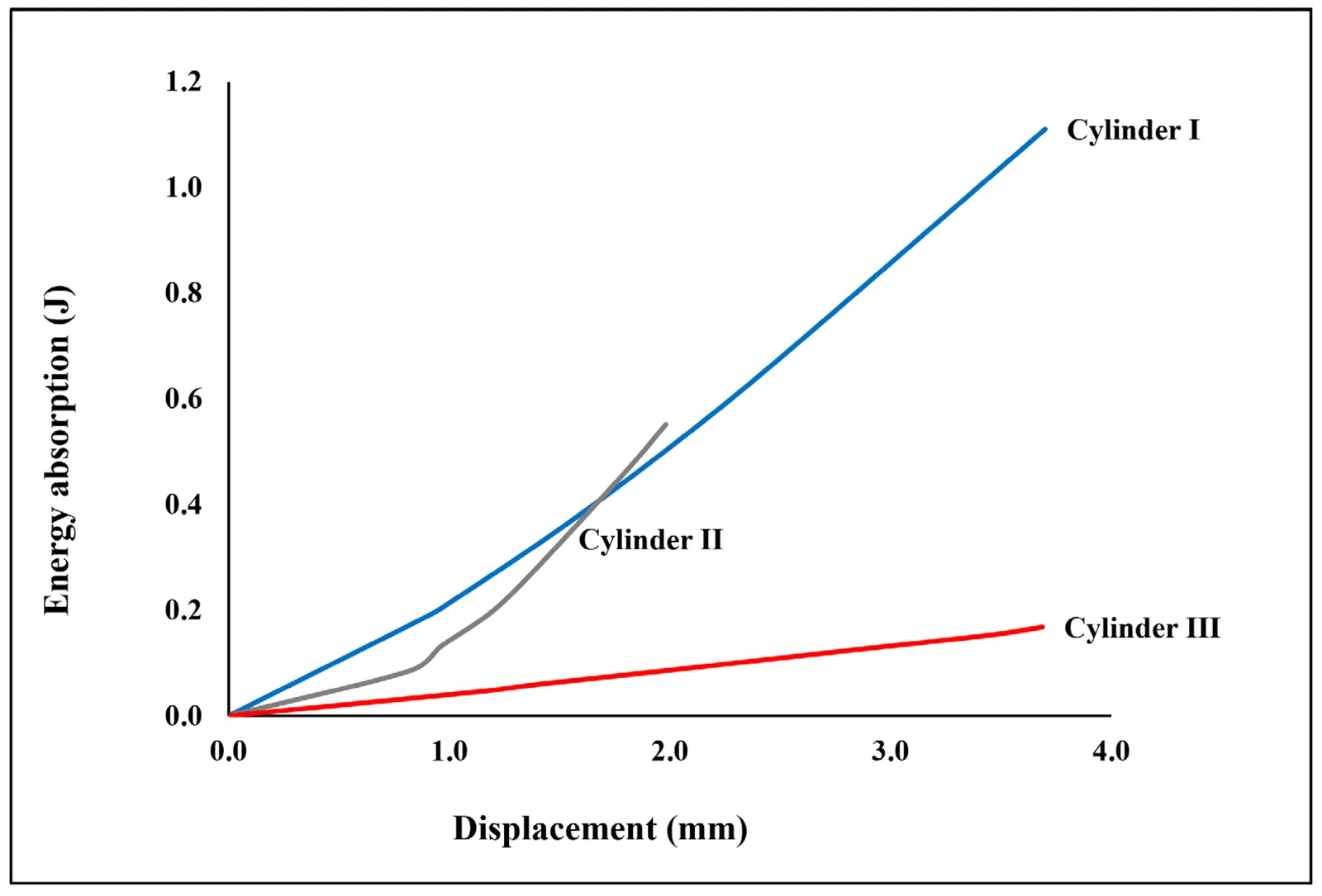
Energy-displacement curve of three Voronoi structures.

Figure 6 presents the relationship between energy absorption and displacement for the three scaffold configurations. The results clearly show that the geometry of the hollow cylindrical struts has a significant impact on their energy absorption capability. For all the tested designs, energy absorption increased progressively with displacement, indicating that deformation energy was gradually accumulated inside the scaffold structure under compression loading. At low displacement levels, there was a substantial increase in absorbed energy. However, when stress increased, other deformation processes like increasing cell-wall bending, local buckling, and structural collapse were activated, resulting in a more pronounced increase in energy absorption. With a maximum value of 1.1102 J at a displacement of 3.7 mm, Cylinder I had the best energy absorption performance among the designs under investigation. In contrast, Cylinder III only recorded 0.1675 J at the same displacement level of 3.7 mm, while Cylinder II absorbed 0.551 J at a displacement of 2 mm. Since Cylinder I has a smaller inner radius and thicker load-bearing struts that can withstand higher loads before failing, it performs better. By delaying the onset of local buckling and increasing wall thickness, more deformation energy can be absorbed prior to collapse, improving structural stability. On the other hand, increasing the inner radius causes the effective wall thickness to decrease, which leads to early instability and a decreased ability to absorb energy.

**Figure 7:**
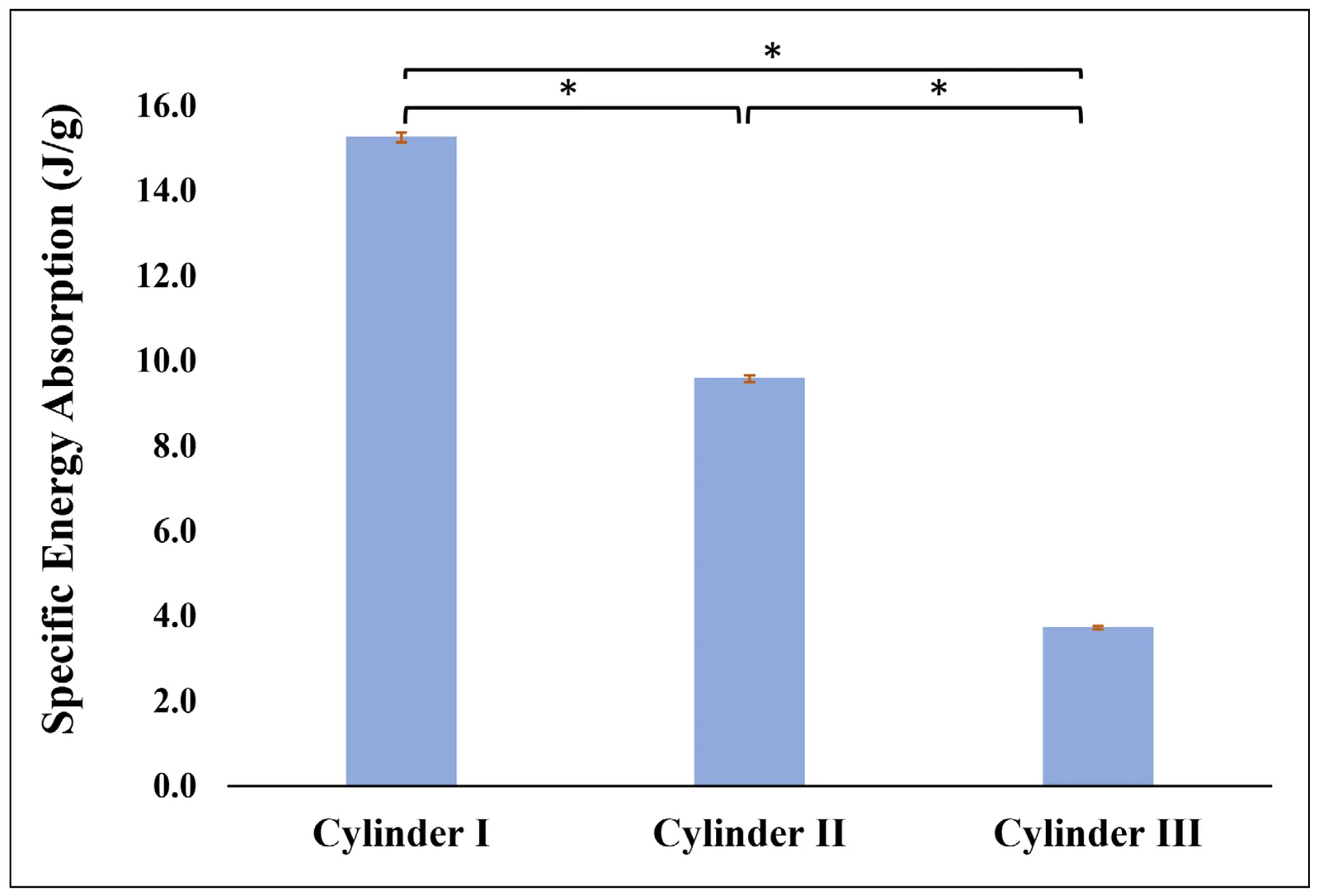
Specific energy absorption (SEA) of three Voronoi structures. Note: Error bars indicate the standard deviation of *n* = 4 tested samples. Statistical Markers: a single asterisk notation (*) denotes a statistically significant difference (*p* < 0.05). Designs with differing letters are statistically distinct.

On the other hand, Figure 7 compares the specific energy absorption (SEA) values of the three scaffold configurations. The error bars represent the standard deviation obtained from four repeated compression tests and are relatively small, describing good experimental consistency. Among all the designs, Cylinder I achieved the highest SEA value of about 15.26 J/g, which is approximately four times greater than that of Cylinder III (3.73 J/g), while Cylinder II exhibited intermediate performance with an SEA of 9.58 J/g. These findings show that lowering the scaffold’s inner radius significantly enhances energy absorption efficiency while not increasing its overall mass. In addition, the superior performance of Cylinder I could be attributed to its thicker hollow strut walls, which provide greater resistance to deformation and allow the structure to absorb more energy before failure. In contrast, increasing the inner radius reduces the effective load-bearing material within each strut, resulting in lower structural stability and a reduced capacity for energy dissipation. Similar trends were reported by Lu et al. [37], who observed that increasing the void percentage in Voronoi cellular structures leads to a drop in energy absorption performance. The obtained SEA values compare favourably with those reported for several lightweight cellular materials, which range from approximately 1 to 10 J/g for aluminium alloy Voronoi structures under impact loading. Therefore, the significant performance achieved by Cylinder I shows the effectiveness of the proposed hollow Voronoi design for enhancing energy absorption while maintaining a lightweight structure. Furthermore, the improved SEA of Cylinder I is also related to the inherent features of the Voronoi cell structures. Previous studies have shown that stochastic Voronoi networks distribute stresses more uniformly and reduce local stress concentrations compared with regular lattice structures [48,49]. Additionally, Zhao et al. [48] showed that Voronoi-based designs are more resilient to impact loading and deformation than traditional porous structures with comparable relative densities. Conversely, the sharp reduction in SEA observed for Cylinder III suggests that excessive extension of the internal hollow structure weakens the energy dissipation mechanisms of the cylindrical Voronoi scaffold. The thinner strut walls become more receptive to local buckling and premature failure, limiting the amount of deformation energy that can be absorbed. Similar observations were reported by Rezapourian and Hussainova [50], who discovered that in hydroxyapatite Voronoi scaffolds, severe structural density decreases had a negative impact on strength and energy absorption performance. From a biomedical perspective, the combination of high compressive strength, high Young’s modulus, and superior SEA obtained for Cylinder I represent a desirable balance between mechanical reliability and lightweight performance. And from a structural–mechanical engineering perspective, the design seeks to maximise the strength-to-weight ratio. Patient-specific customisation is made possible by the inner strut radius. Higher-strength designs (Cylinder I) better sustain denser bone formations, whereas lower-stiffness designs (Cylinder III) are appropriate for osteoporotic bone.

## 4. Conclusions

This study investigated the effect of the inner diameter of hollow cylindrical Voronoi scaffolds on their mechanical properties and energy absorption performance. The results showed that increasing the inner diameter from 0.5 mm to 1.5 mm significantly reduced the elastic modulus, ultimate compressive strength, and stiffness. Cylinder I (r = 0.5 mm) exhibited the best mechanical performance, with an elastic modulus of 201 MPa and an ultimate compressive strength of 22 MPa. In contrast, Cylinder III (r = 1.5 mm) showed an approximately 80% reduction in stiffness. Cylinder I also achieved the highest specific energy absorption (SEA), which was nearly four times greater than that of Cylinder III.

The reduction in mechanical properties is mainly attributed to the thinner strut walls at larger inner diameters, where these thinner walls are more susceptible to stress concentrations and local buckling, leading to earlier structural failure under compression. Overall, the findings demonstrate that controlling the inner diameter is an effective method for balancing lightweight design with mechanical stability. Combined with the use of eco-friendly, plant-based resin, the proposed Voronoi-based scaffold structure offers a promising design for biomimetic bone tissue engineering applications. Future work will incorporate Finite Element Analysis (FEA) to investigate the local stress distribution within the Voronoi structures and support further design optimisation.

## Figures and Tables

**Table 1:** The geometry properties of the three models.

Property	Cylinder I	Cylinder II	Cylinder III
Seeds	116	125	125
R (mm)	3.27	3.27	3.27
H (mm)	12	12	12
Weight (g)	0.2	0.19	0.19

**Table 2:** Mechanical properties of models.

Property	Unit	Cylinder I	Cylinder II	Cylinder III
E *	MPa	201 ±0.94	142 ±0.66	42 ±0.20
S *	N/mm	368.4 ±1.36	286.9 ±1.06	42.9 ±0.16
US	MPa	22±0.01	16.3 ±0.11	4 ±0.03

* Asterisk (*) denotes apparent values.

**Table 3:** Comparison of the mechanical properties of the proposed hollow Voronoi scaffolds with recently reported bone tissue engineering scaffolds.

Design	Porosity (%)	Relative Density (%)	E* (MPa)	US (MPa)	Reference
Cylinder I	63	37	201	22	Current Study
Cylinder II	63	37	142	16.3	Current Study
Cylinder III	63	37	42	4	Current Study
Diamond and Gyroid	65	-	85–135	4–5.5	[42]
Schwarz-P and Gyroid	~50	-	60–90	9.8–11.3	[43]
I-WP Lattice	60	40	35	1.93	[44]
	50	50	36	2.29	
Primitive and Lattice Diamond and Gyroid	70.1–81.8	18–30	~700–3400	~1–4.2	[45]

* Asterisk (*) denotes apparent values.

## Data Availability

The original contributions presented in the study are included in the article; further inquiries can be directed to the corresponding author.

## Appendix A

The Rhinoceros 7 (Grasshopper) software was implemented to generate topologies for the three designs for Voronoi structures. Figure A1 shows the Grasshopper file for the hollow Voronoi structures (HVSs). HVSs were generated by seeding random points using the two plugins (Populate 3D Geometry and 3D Voronoi). Then a Bounding Box plugin was created to assist in aligning the Voronoi structure within the cylinder. The Solid Intersection and Deconstruct Brep plugins were added to enclose the cells in the required volume. Followed by adding the Pipe plugin to achieve the hollowing effect with specific radius parameters. The plugins are the same for the three designs, with just the radius being controlled by the Slider number—green highlight—that is connected to the geometry (cir).
